# The Institutional Worker

**Published:** 1921-02-19

**Authors:** 


### THE INSTITUTIONAL WORKER.

*Being a Special Supplement to "The Hospital."*

### OUR BUREAU OF INFORMATION.

### INSTITUTION FACTS AND FIGURES.

### The Question Box.

### CHECKS ON WASTE.

### Answer to January Question.

THE question was :

**Describe methods for keeping a check on the use of drugs and dressings in the hospital wards and operating-theatre with a view to prevent waste.**

**"Odd No." replies:**

There is probably no branch of hospital work which presents more acute or more varied problems than that of the dispensary department of a large hospital.

The method of issue of drugs and dressings to all departments of a hospital, as outlined below, has proved itself efficient, and the slight initial outlay in requisition books, etc., has been more than counterbalanced by the saving in consumption.

All dressings, etc. (excluding drugs), are requisitioned twice weekly by each department, theatre, or ward in a book ruled as shown below :—

Ward

Week ending ———— 19

| — | Quantity | | Total | Rate | Total |
|---|---|---|---|---|---|
| | | | | | £ |
| Bandages, 1½" ... | | | | | |
| " 2½" ... | | | | | |
| " 3" ... | | | | | |
| " 4" ... | | | | | |
| Lint ... ... | | | | | |
| Gauze ... ... | | | | | |
| &c.... ... ... | | | | | |

Total : £

Average Daily number of Patients :—

Please supply above ———— Sister

Issued ———— Dispenser

Received ———— Sister

House Governor or Secretary.

Each sheet shows a week's requisitions, the two "quantity" columns providing space for the entry of each of the half-weekly requirements. Each weekly page in the dressings requisition book is signed by the sister in charge of the ward or department as the requisition is made, and again on receipt of the dressings from the dispensary. The dispenser fills in the cost of the several articles, and the books are then sent each week for signature by the House Governor or Secretary and inspection by the Committee.

By this means an accurate record is kept of the quantities and costs of all dressings used, and the consumption of one ward or department may conveniently be compared with that of another ward or department. At the end of each month the total amount of dressings used is entered, ward by ward, on a monthly summary sheet, the figures, of course, being easily ascertainable from the dressings books themselves. A specimen of the comparative return is given below, from which it will be seen that the return may be extended to include stationery, crockery, washing, and other items of expenditure :—

*Monthly Summary of Ward Costs, January 31, 1921.*

| Ward No. or Department | No. of Beds | Drugs | Dressings | Crockery and Hardware | Stationery | Washing | Total |
|---|---|---|---|---|---|---|---|
| O.P. Dept. ... | | | | | | | |
| Casualty ... | | | | | | | |
| X-Ray ... ... | | | | | | | |
| Ward 1... ... | | | | | | | |
| Ward 2 .. ... | | | | | | | |
| &c. ... ... | | | | | | | |

Copies of this comparative return are handed to the matron, the medical staff, and also to the sister of each ward or department. This system engenders a healthy spirit of competition amongst the sisters and nurses, ward against ward. Any excessive consumption is at once detected, and the sister or person responsible is called upon by the committee to explain the reason of any abnormal increase in a particular department of the hospital.

With regard to the supply of drugs, etc., to the wards and theatres, this is done by means of drug requisition books ruled in the same way as the dressings requisition books, but with the names of the drugs printed down the left-hand side, with the addition of a few extra lines for special prescriptions.

The drug requisition books are entered up by the ward sister from the actual prescriptions made by the medical staff on each individual case paper. This work should be done daily and the books sent to the dispensary for the supply of drugs. The cost of drugs issued may conveniently be entered by the dispenser weekly, or more often as the peculiar requirements of any particular institution may necessitate. The drug requisition books are, of course, subject to periodical inspection by the committee in the same way as the dressings requisitions. Emergency requisitions for drugs, etc., may be made in duplicate books with tear-off leaves signed by the sister. At the end of each day all such emergency orders should be added to that day's regular supplies.

All unused drugs, dressings, etc., should be handed each day to the sister of the ward or department for returning to the dispensary or kept in stock against future requirements. The sister should, on her daily visits to each patient, ascertain as far as possible if there is any waste in the administration of drugs or dressings, and should see that the nurses are economical in the use of bandages and expensive drugs.

A very frequent cause of considerable waste in the wards arises from the fact that patients' friends often bring to the ward drugs, Virol, Bovril, eggs, etc., thus duplicating the supplies from the dispensary and kitchen departments of the hospital. To obviate this waste an accurate record should be kept of all articles brought to the patient by friends. The ward sister should see that in all cases such gifts are deducted from the requisitions to the dispensary or kitchen.

Losses of dressings and instruments frequently arise when the sister in charge of a ward or department leaves the service of the hospital without making an accurate record of the stocks on hand. It should be the duty of the matron or the dispenser to see that a complete inventory is taken whenever a change takes place in the sister's post, as a new officer cannot reasonably be expected to be held responsible for any shortage occurring before her appointment. The adoption of this method has proved the means of considerable saving in expense, especially in relation to surgical instruments, etc.

The system outlined above, with modifications to suit the peculiar requirements of any individual institution, may prove the means of vastly reducing the expenditure under "Surgery and Dispensary"—more especially if supported by a keen dispenser and a loyal nursing staff.

### Question for February.

### HYGIENE INSTRUCTION AT WELFARE CENTRES.

**Outline a scheme for the collective instruction in hygiene of expectant and nursing mothers, and also of voluntary and salaried workers, at a Maternity and Child Welfare Centre. The expenses of such instruction being provided for by the grant offered for this purpose under the Maternity and Child Welfare Act 1918.**

**(A minimum payment of 10s. 6d. will be made for each published answer.)**

RULES.

The following rules must be observed:—

1. Contributions must be written on one side of the paper. Conciseness and terseness are desirable features. The MS. must bear the name and address of the sender and be accompanied by coupon to be cut from the back cover (inside page) of the current issue of The Hospital. A pseudonym must be chosen if the name is not to be published.

2. Contributions must be addressed to the Editor of The Hospital Institutional Worker Supplement, 28 & 29 Southampton Street, Strand, London, W.C. 2, should reach him before the end of the current month, and be marked in the left-hand corner "Facts and Figures."

### *THE SICK AND IN NEED.*

### Home Required for Aged Tradesman.

Could any of our readers suggest a suitable Home in or near London for a retired tradesman, aged seventy-five. His health is good, but he requires rest and a little attention. He could contribute about £60 a year towards his support.

### Treatment for Rheumatism in Bath.

We suggest that you write to the Secretary of the Royal Mineral Water Hospital, Bath, and ask if you are eligible for admission to that institution. If you are not eligible, perhaps the Secretary will suggest some private institution that would be within your means.—M. E. H.

### Dispensary in Brighton.

The Brighton, Hove, and Preston Dispensary is at 113 Queen's Road, Brighton. Perhaps the Hon. Secretary will assist you in obtaining a letter to enable you to attend the dispensary. A registration fee of sixpence is all that is necessary upon presenting each letter.—T. R. T.

### Convalescent Home in Cornwall.

Write for a vacancy to the Hon. Secretary of the Cornwall Convalescent Homes for Men and Women, Perranporth. Admission is free by letter of subscriber, by hospital or union recommendation and payment of 12s. 6d. weekly, or by payment of £1 weekly. A letter lasts two weeks.—Budleigh.

### *EMPLOYMENT AND TRAINING.*

### Voluntary Aid Workers.

Please read the rules. We do not give postal replies. Your experience in the Voluntary-Aid Detachment should enable you to obtain the occupation you desire. We would suggest that you communicate with the Commandant of the detachment you previously worked under. She could doubtless help you in this direction. As an alternative you might write to the matrons of some of the general hospitals.—F. S.

### Training Required for Sister-Tutorship.

The Secretary of King's College for Women, Campden Hill, W., will send you, upon application, full particulars of training required for Sister-Tutorship.—K. S. W.

### *MISCELLANEOUS.*

### Entertainment of Wounded Soldiers.

There are few, if any, Service men accommodated in the voluntary hospitals in London. Your best course would be to write to the Deputy-Director of Medical Service, London District, Horse Guards, S.W. 1. You might also make your offer to the Director of Medical Service, Ministry of Pensions, Millbank, S.W., to the British Red Cross Society, 19 Berkeley Street, W. 1, and to Miss Marta Cunningham, The Not-Forgotten Association, 86 Ladbroke Road, W. 11. A coupon should be enclosed with each inquiry. We do not give postal replies.—E. H. R.

